# A meta-analysis and experimental data for multidrug resistance genes in breast cancer

**DOI:** 10.4314/ahs.v22i4.2

**Published:** 2022-12

**Authors:** Shumaila Zaib, Sammia Tahir, Nosheen Masood, Abdul Hameed, Yasmin Azra

**Affiliations:** 1 Microbiology and Biotechnology Research Lab, Department of Environmental Sciences, Fatima Jinnah Women University, Rawalpindi, Pakistan; 2 Microbiology and Biotechnology Research Lab, Department of Biotechnology, Fatima Jinnah Women University, Rawalpindi, Pakistan; 3 Institute of Biomedical and Genetic Engineering, Islamabad, Pakistan

**Keywords:** Expression, mutations, mdr1, ABCG2, breast cancer

## Abstract

**Background:**

Increasing trend of breast cancer incidence worldwide is a known fact. This curable disease may become fatal if drug resistance is developed leading to metastatic cancerous tissue.

**Objective:**

This is a two parts study; a meta-analysis exploring association of drug resistance (mdr1 and ABCG2) genes with breast cancer and mutational association with molecular subtypes of cancer. Methods: PCR-SSCP for genomic polymorphisms and RT-PCR for expression analysis were performed.

**Results:**

C3435T polymorphism of mdr1 gene was most commonly studied mutation with contradictory results. Association of ABCG2 gene mutations with untreated breast cancer was reported only by one study so far. Regarding current genomic analysis of mdr1 gene, three novel mutations were found in exon 12 and 2 mutations were found in exon 26. In ABCG2 gene, addition of C and T were found in intron 8 at the intron-exon junction. A positive correlation was observed between these mutations and tumor grade. Levels of mRNA expression revealed that they were over expressed in cancerous tissues compared with controls.

**Conclusion:**

These findings suggest that these genes are associated with breast cancer.

## Introduction

Breast cancer is the second most common cancer in US as well as in Asian countries^1^, while in Pakistan 1/9 suffer from breast cancer^2^. Initiation of breast cancer cells often starts undetected through lymph channels, contiguity and blood. At latter stages of breast cancer, the most common sites that become metastatic are bones, lymph nodes, liver, skin, lungs and brain^3^. Combinations of multiple complex extrinsic (environmental) and intrinsic (hormones, epigenetic and genetic) factors are responsible for initiation and propagation of breast cancer.

One of the most common issues faced by oncologists in treating breast cancer is the drug resistance. Genes involved in ABC transporters are ATP dependent efflux pumps and are responsible for adsorption, distribution, metabolism, excretion and transportation of anticancer drugs towards cancer cells. One of these genes is mdr1, which is an ATP-dependent efflux transporter, and protect body from environmental toxins. It functions to reduce the intracellular concentration of a wide spectrum of drugs and antibiotics^4^. It has been suggested that single nucleotide polymorphisms (SNPs) of mdr1 could influence the level of expression of enhancer and promoter sequences which may influence the efficacy of processing of the pre-mRNAs, and consequently mRNA stability^5^. Among more than 50 SNPs identified in ABC gene, the most clinically relevant SNPs are C3435T (rs1045642) in exon 26, G2677A/ T in exon 21 (rs2032582) and C1236T (rs1128503). Synonymous SNPs (C3435T and C1236T) decrease the levels of mRNA expression^6^, whereas, G 2677A/T leads to change of alanine amino acid with serine or threonine. Many researchers discussed the relation between the three SNPs and the incidence of breast cancer^7^, ^8^. However, the findings of these studies are still inconclusive 8. When it is overexpressed in breast cancer, it causes drug resistance of cancer cells to drugs given for breast cancer therapy.

Another ABC transporter gene is ABCG2, which is an ATP binding cassette membrane transporter and is associated in the control of absorption, distribution and clearance of numerous xenobiotics, including dietary carcinogens, pharmaceutical agents and conjugated metabolites. ABCG2 gene is located on chromosome 4q22 and encodes a 655-amino acid protein. Single-nucleotide polymorphisms (SNPs) in the ABCG2 gene have been identified in various ethnic populations^9^. The most frequent SNPs in the ABCG2 gene are G34A (rs2231137, V12M), and C421A (rs2231142, Q141K). These polymorphisms are associated with decrease in expression as well as transporter activity of the ABCG2 protein^10^. An overexpression of ABCG2 has been found in drug resistant breast cancer tumor cells.

Present study was designed to observe the mutational spectrum and mRNA expressional variations of mdr1 and ABCG2 and its association with different environmental, clinical, and histopathological parameter in breast cancer patients in Pakistani population. Initially a meta-analysis was performed involving previous studies and then the results were compared to obtain a clear picture about the role of these genes in breast cancer.

## Materials and methods

### Selection strategy for meta-analysis

Literature search, study design and data analysis were performed following PRISMA (Preferred Reporting Items for Systematic Reviews and Meta-Analyses) guidelines (see Supplemental Contents—PRISMA checklist (http://links.lww.com/MD/B316). A detailed literature search was conducted for journal article using Pub Med database for all eligible studies reporting mdr1 and ABCG2 polymorphism/mutations and mRNA expressional variations using different search words (breast cancer, mdr1, ABCG2, polymorphisms, mutations, genetic variations, expressional variations, mRNA, etc.). There was no restriction on sample size, ethnicity of population, language. All eligible studies were retrieved and checked for other relevant studies. The literature retrieval was performed in duplication by two independent reviewers. Studies were included only if they met the following criteria:^1^ case-control studies which evaluated the association between mdr1 or ABCG2 polymorphisms and breast cancer risk; 2 studies using any of the mutation detection techniques (e.g., PCR-RFLP, PCR-SSCP, ARMS-PCR, qRT-PCR); 3 studies published as full articles in English. A number of studies were excluded on the basis of the following points.^1^ Studies using cancer cell lines, tumor samples, serum, or saliva samples were not included;^2^ Review articles and previous meta-analysis were also not included;^3^ Studies on diseases other than cancer were also excluded from present study. Screening of eligible records and selection of articles to be included in the meta-analysis were independently performed by 2 reviewers and disagreements were resolved by discussion and consensus.

### Sampling

Total 25 breast cancer tumor samples and 25 control tissues were collected from patients undergoing surgery at Pakistan Institute of Medical Sciences Hospital, Islamabad before chemotherapy. Study started after approved from ethical committees of concerned hospitals and university while samples were collected after getting signed informed consents from patients. Freshly excised tissue samples along with surrounding normal tissue were collected in RNA later solution and stored at -80°C freezer at Institute of Biomedical and Genetic Engineering, Islamabad for further use. Data regarding age, marital status, menarche, menopause, and disease onset was collected from patients and stage/ grade was extracted from pathological findings.

### Genome Analysis

DNA was extracted from breast tissues by organic method^11^ followed by PCR amplification with specific primers and SSCP^12^ suspected samples were sequenced. Each PCR reaction was performed in a 50L reaction mixture containing 5*µ*L of genomic DNA templates, 2*µ*L of each primer, 30*µ*L nuclease-free water, 5*µ*L 10X buffer, 3 *µ*L of 25mM MgCl2, 4*µ*L of 2.5 mM dNTPs, 1*µ*L Taq polymerase (Thermo Scientific). Initial denaturation of reaction mixture was done at 94°C for 1 minute in a single cycle, followed by 35 cycles of 3 step PCR comprised of 94°C for 45 sec, exon specific annealing temperature for 45 sec and extension at 72°C for 45 seconds. It was followed by a final extension step at 72°C for 10min and then stored at -20°C until further processing. 2 *µ*L of PCR products along with loading dye were electrophoresed on a 2% agarose gel and stained with ethidium bromide. 100 bp ladder was also loaded as standard for quantification of amount and confirmation of PCR product size. Single stranded conformational polymorphism (SSCP) was performed for identification of mutated samples. Selected samples showing altered mobility were sequenced commercially (Macrogen, Korea). Results were analysed by bioedit software.

Total RNA extraction was done by Trizol method according to manufacturer's protocol (Invitrogen, California, USA). After extraction RNA was quantitatively analyzed by NanoDrop (Thermo scientific 2000 UV-spectrophotometer) and qualitatively by gel electrophoreses on a 1.5% TAE agarose gel. Total RNA was converted into cDNA by using SuperScriptTM III First-Strand Synthesis Kit (Invitrogen Cat# 18080-051). Reverse Transcriptase PCR was used to amplify the sequence of mdr1 and ABCG2 cDNA (table 1). Reaction mixture was initially denatured at 94°C for 2 minute, followed by 40 cycles of denaturation at 94°C for 15 seconds, primer specific annealing temperature for 30 seconds, extension at 72°C for 1 minute, final extension was done at 72°C for 10 minutes and then hold at temperature 4°C. TBE agarose gel electrophoresis (2%) was performed to analyse expressional variations according to the method described by 13.

**Table 1:** Forward and reverse primer sequences for Mdr1, ABCG2 and β-actin mRNA with their annealing temperatures

Genes	Forward Primer	Reverse Primer	Annealing Temperature
**MDR1**	CAGAGGCTCTATGACCCCAC	CTTCTGCCCACCACTCAACT	58 °C
**ABCG2**	GGTGGAGGCAAATCTTCGTT	AGCCAGTTGTAGGCTCATCC	58 °C
**β-actin**	GCTCGTCGTCGACAACGGCT	CAAAACATGATCTGGGTCATCTTCTC	55°C

## Results

### Meta-analysis

The data search retrieved 40 full length articles and after applying inclusion exclusion criteria only 12 studies were included for mdr1 in association with breast cancer 14–25. Among them 13 studies were excluded as they were on other cancers except breast, 7 reports involved cell cultures rather than tissue or blood, 3 reports were review or meta-analysis and 5 studies included just healthy population without any disease. Altogether 2044 individuals were included in meta-analysis (1165 breast cancer patients and 879 controls) (table 3). Among them 7 studies used PCR RFLP and 5 used RT PCR techniques for screening. Total 7 studies showed expressional variations in mdr1 gene, 7 studies were on mutational analysis and 2 studies reported both mutational and expressional variations. Among mutational analysis; C3435T mutation was studied by 6 studies, 1 study focused on A412C mutation while another involved G2677A/T and C1236T mutations along with C3435T mutation. Regarding mutational analysis of C3435T mutation contradictory reports have been observed and found that 2 studies involving 207 patients showed association of C3435T mutation with breast cancer whereas 4 studies including 263 patients had no association of this mutation with breast cancer. Total 22 studies were found on ABCG2 mutations. Among them 15 were excluded due to their focus on normal population or any other disease than breast cancer, while 6 studies involved a response to particular drug. Only 1 research article was found for ABCG2 gene involving 1169 breast cancer patients from China, screened for mutational analysis using PCR-RFLP technique 26.

**Table 3:** Review of literature added in the meta-analysis for mdr1 gene showing all the details

Ref	Year	Tumor description	Treatment description	mdr1 gene mutation	Association	mdr1 mRNA expression	Number of controls	Number of patients	Methodology used	Country
**(14)**	2016	breast cancer	No	C3435T, G2677A/T, C1236T	C3435T, G2677A/T, but not C1236T	No	150	150	PCR RLFP/ Sequencing	Jordan
**(15)**	2003	primary breast cancer	Chemotherapy	no	no	yes	no	59	Real time RT PCR	Netherland
**(16)**	2001	breast cancer	Chemotherapy naïve	no	no	yes	no	52	Real time RT PCR	Netherland
**(17)**	2014	primary breast cancer	before treatment	C3435T	no association	no	183	64	PCR RFLP	Mexico
**(18)**	2013	breast cancer	no	A412C	no association	no	348	340	PCR RFLP	China
**(19)**	2012	breast cancer lymph nodes	no	no	no	higher	21	21	RT PCR	China
**(20)**	2011	breast cancer	no	C3435T	no association	higher	no	39	PCR RFLP immunohistochemistry	Slovak Republic
**(21)**	2001	advancebreast cancer	Chemotherapy	no	no	associated	no	52	RT PCR	Spain
**(22)**	2010	Primary breast cancer	No treatment	C3435T	no	associated	50	54	PCR RFLP and Real time QPCR	Iran
**(23)**	2009	Ductal/ lobular breast cancer	Any treatment	C3435T	no association	no	77	106	PCR RFLP	Iran
**(24)**	2007	invasive ductal cancer	Any treatment	C3435T	associated	no	50	57	PCR RFLP	Turkey
**(25)**	2006	invasive breast cancer	Any treatment	no	no	no association	No	171	RT PCR	France

### Genome analysis

Second part of study involved mutational analysis of mdr1 and ABCG2 genes. For this purpose, 25 breast cancer tissues and 25 normal breast tissues were collected and were amplified via PCR followed by SSCP. The mean age of patients was found to be 46.4 + 11.2 years and 24% breast cancer patients had a family history of breast and ovarian cancer. Clinico-pathological data has been presented in table 2. SSCP was used followed by sequencing of suspected samples. Three novel mutations; two substitutions (g.1565A>T and g.1571A>T) and one deletion (g. 1567delC) were observed in exon 12 of mdr1 gene and 12% patients and one control had these mutations. These mutations changed the amino acid sequence at position 413 from leucine to tyrosine and the deletion of C resulted in frameshift mutation. Among the patients showing these mutations, 60% were at 3rd stage of breast cancer. These mutations were found to be significantly associated (P<0.05) with breast cancer and a ∼5 folds (OR=4.5; CI 0.5–44.2) increased chances of mutations are present in case of breast cancer compared with controls.

**Table 2:** Frequency of different clinico-pathological characteristics of Breast cancer patients

Characteristics	Percentage	
Stage I-II	92%	
Stage III-IV	8%	
Positive cancer family history	25%	
Marital status	Single	12%
	Married	88%
No. of children	Nulliparous	6%
	2 to 4	62%
	5 to 8	32%
Children on breast feeding	Yes	71.5%
	No	28.5%
Early menarche	Before 13 years of age	37%
Menopause	Before 45 years of age	63%
Chemotherapy Sessions	Zero	40.19%
	1 to 3	42.16%
	4 to 5	17.65%
Lump in breast		40%
Nipple retraction		15%
Change in nipple size		12%
Bleeding		8%
Dense or hard mass in breast		25%
Onset of disease respect to age		
25 ≥ 40		32%
40 > 60		56%
61 and above		12%

Two different types of mutations were observed in exon 26 involving one reported C3435T mutation (rs1045642) and one novel addition of A at position 3664. It was found that 12% tumor samples showed C3435T mutation and was significantly associated (P<0.05) with breast cancer risk compared with controls. Approximately 75% of samples showing this mutation were froadvanced (3rd or 4^th^) stage of cancer. It was found that this mutation has ∼3 folds chances of occurrence in breast cancer patients compared with controls (OD=3.4; CI 0.3–35.5). Similarly, 4% patients revealed addition of A resulting in frameshift mutation at position 1113 in amino acid sequence and no normal sample showed this mutation revealing highly significant association (P<0.05) with breast cancer. All the mutations were rechecked with reverse primer as well for confirmation.

In ABCG2 gene, two intronic mutations were found at the intron exon junction upstream of exon 9. Both mutations involve additions; at position 88,113,522 C and at position 88,113,525 another T was added. These mutations might hinder in exon splicing and lead to mutated protein structure. Approximately 32% patients showed these mutations and the mean age of patients having these mutations was 67+1.3 years. These mutations were found significantly associated (P<0.05) with breast cancer with an approximately 5-fold increased risk of breast cancer (OD=5.4; CI 1–28.8) compared with controls.

**Figure 1:**
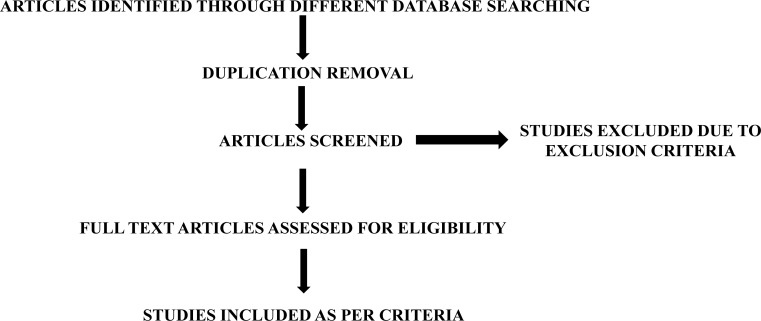
A schematic flowchart showing the method of assessing research articles for metanalysis.

Expression of mdr1 and ABCG2 genes in mRNA was checked along with beta actin as house-keeping gene (Fig 1). The results showed that both the genes were over-expressed in case of breast cancer tissue compared with controls. Approximately 44% and 32% cancerous tissues overexpressed mdr1 and ABCG2 respectively, showing a significant association (P = 0.0113 and P = 0.02) with cancer risk. A non-significant (P>0.05) over expression of both the genes was observed in rest of the 24% patients.

Most of the samples showing mutations in DNA also showed lower expression of mdr1 and ABCG2 genes compared with their expression in other cancerous tissue samples. These results revealed that mutations at DNA level may have some effect on its mRNA expression as well.

## Discussion

Breast cancer has been increasing at an alarming rate in Pakistani population and the exact etiology is still unknown. mdr1 and ABCG2 are two important genes of ABC transporter pathway that are thought to be mutated and ultimately leading to cancer initiation. Another important role of these genes is in drug resistance which has given prime importance in the treatment of cancer patients. Mutations or abnormal expression of these genes can cause severe drug resistance and patients may not respond to the drug given to them as treatment. Many reviews have been published up till now discussing the importance of mdr1 and ABCG2 genes but the results have been contrasting regarding its association with breast cancer and no data is available in Pakistani population. Moreover, studies with comparatively larger sample size are required for exploring the association of these gene mutations with clinico-pathological parameters and drug resistance. The results of current meta-analysis revealed that C3435T mutation in mdr1 gene was most frequently studied 27, only one study reported other mutations along with this in breast cancer patients. Unfortunately only one report was found detailing the mutational analysis of ABCG2 gene with breast cancer however its association with other cancers has been reported in many studies^28^. Therefore, this study is of prime importance to know the mutational pattern of ABCG2 gene among Asians.

In the second part of study regarding genomic and mRNA expressional variation in mdr1 and ABCG2 genes interesting results have been found. Three novel mutations in mdr1 gene exon 12 including g.167968A<T, g. del167970C & g.167973A<T while in exon 26 one already reported (C3435T) mutation and one novel mutation (g. add1113A) was found in the current study. All these mutations were significantly associated with breast cancer compared with controls. Similar studies have been carried out in many different cancers and novel as well as already reported mutations were found. Most of these studies were reviewed by Wang et al.^29^. In ABCG2 gene, two additions of C and T were found in intron 8 most of the studies have focused on association of ABCG2 gene with colon cancer and have found novel mutations 30. Mutations found in current study needs to be further explored for its exact role in mRNA expression.

Levels of mRNA have been found to be elevated in case of breast cancer compared with normal tissues. The results are in accordance with reported literature^30^. Over expression of mdr1 and ABCG2 genes may not always be indicative of cancer initiation, in pancreatic cancer lower expression correlates with cancer initiation^31^, but in this study significant association of mRNA expressional variation of mdr1 and ABCG2 genes have been found with breast cancer risk. Normal disease free population of Pakistan has also been screened for ABC transporter genes and frequent C3435T mutation was found however their genotypic frequencies are different than rest of the Asian population^32^, ^33^.

In conclusion, mdr1 and ABCG2 genes are frequently mutated in breast cancer patients compared with controls and mRNA expression has been altered due to these mutations. Such results have not been available in Asian population. Most of the studies on these genes have evaluated the effect of mutations with particular drug therefore this research is important in providing a clear picture of genetic and expression status of mdr1 and ABCG2 genes in breast cancer before any drug was administered.

## Figures and Tables

**Figure 2:**
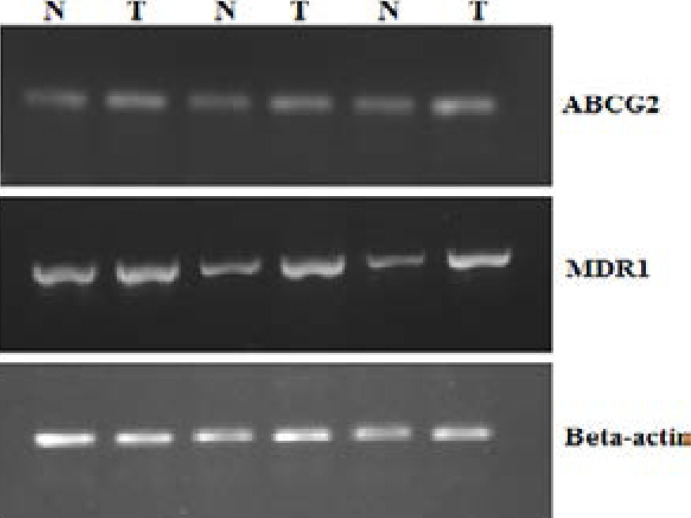
A 2% agarose gel electrophoresis showing expression of mdr1, ABCG2 and beta actin in tumour and normal tissues. Sample numbers are shown above alphabets, N shows normal and T shows breast cancer tissues.
